# Functional Interplay between the 53BP1-Ortholog Rad9 and the Mre11 Complex Regulates Resection, End-Tethering and Repair of a Double-Strand Break

**DOI:** 10.1371/journal.pgen.1004928

**Published:** 2015-01-08

**Authors:** Matteo Ferrari, Diego Dibitetto, Giuseppe De Gregorio, Vinay V. Eapen, Chetan C. Rawal, Federico Lazzaro, Michael Tsabar, Federica Marini, James E. Haber, Achille Pellicioli

**Affiliations:** 1 Department of Biosciences, University of Milan, Milano, Italy; 2 Department of Biology and Rosenstiel Basic Medical Sciences Research Center, Brandeis University, Waltham, Massachusetts, United States of America; University of Illinois College of Pharmacy, United States of America

## Abstract

The Mre11-Rad50-Xrs2 nuclease complex, together with Sae2, initiates the 5′-to-3′ resection of Double-Strand DNA Breaks (DSBs). Extended 3′ single stranded DNA filaments can be exposed from a DSB through the redundant activities of the Exo1 nuclease and the Dna2 nuclease with the Sgs1 helicase. In the absence of Sae2, Mre11 binding to a DSB is prolonged, the two DNA ends cannot be kept tethered, and the DSB is not efficiently repaired. Here we show that deletion of the yeast 53BP1-ortholog *RAD9* reduces Mre11 binding to a DSB, leading to Rad52 recruitment and efficient DSB end-tethering, through an Sgs1-dependent mechanism. As a consequence, deletion of *RAD9* restores DSB repair either in absence of Sae2 or in presence of a nuclease defective MRX complex. We propose that, in cells lacking Sae2, Rad9/53BP1 contributes to keep Mre11 bound to a persistent DSB, protecting it from extensive DNA end resection, which may lead to potentially deleterious DNA deletions and genome rearrangements.

## Author Summary

DNA double strand breaks (DSBs) are among the most deleterious types of damage occurring in the genome, as failure to repair these lesions through either non-homologous-end-joining (NHEJ) or homologous recombination (HR) leads to genetic instability. The 5′ strand of a DSB can be nucleolytically degraded by several nucleases and associated factors, including Mre11, CtIP/Sae2, Exo1 and Dna2 together with Bloom helicase/Sgs1, through a finely regulated process called DSB resection. Once resection is initiated, error-prone NHEJ is prevented. Several findings suggest that DSB resection is a double-edged sword, if not finely regulated, since on one hand it is needed for faithful HR, but on the other it may lead to extensive DNA deletions associated with genome instability. Both in mammals and yeast, 53BP1/Rad9 protein binds near the lesion and counteracts the resection process, limiting the formation of ssDNA. By using *S. cerevisiae* as a model organism, here we show that Rad9 oligomers block the removal of hypo-active Mre11 protein from a persistent DSB, thus limiting initiation of resection and the recruitment of the recombination factor Rad52, in the absence of Sae2. Altogether, these findings pinpoint a critical role of 53BP1/Rad9 in balancing HR and NHEJ repair events throughout the cell cycle.

## Introduction

Similarly to what is seen in higher eukaryotes, in *S. cerevisiae* the ends of a double-strand DNA break (DSB) are recognized and bound by the Mre11-Rad50-Xrs2 (MRX) complex and the Ku70-Ku80 heterodimer, which compete for end binding. Once the MRX complex, together with CDK1-phosphorylated Sae2 (CtIP in human), initiates resection of the DNA ends, Ku70-Ku80 binding and NHEJ (non-homologous end-joining) are prevented [1], [2], [3], [4]. Subsequent 5′–3′ long-range resection can then occur by one of two pathways: the first utilizes the RecQ helicase Sgs1 (BLM in human), in cooperation with the endonuclease Dna2, and the second utilizes the exonuclease Exo1 [5], [6], [7], [8], [9].

The regulation of DSB end resection is very important to choose the right pathway to repair a DSB and avoid chromosomal rearrangements [10], [11]. Whereas classical NHEJ requires little or no resection, HR (homologous recombination) is characterized by extensive exonucleolytic degradation of one strand. Blocking DNA end resection affects the efficiency and accuracy of how a DSB is repaired. For example, inhibiting resection leads to de novo telomere addition, and eventually loss of a portion of a chromosome [12], [13]. On the other end, extensive DNA end resection could lead to accumulation of unstable DNA intermediates and eventually to the highly error-prone microhomology-mediated end joining (MMEJ) and single-strand annealing (SSA) events, which may cause DNA deletions and translocations [14], [15], [16].

It is now clear that the DNA damage checkpoint response (DDR) plays a central role in regulating DSB end resection. In fact, while resection proceeds, the formation of RPA-coated ssDNA activates the upstream kinase Mec1 (ATR in mammals) and the effector kinase Rad53 (Chk2 in mammals), which in turn phosphorylates and inhibits Exo1 [17]. Interestingly, Exo1 is regulated through a DDR pathway in human cells, too [18], [19].

Moreover, studies both in yeast and mammals showed that Exo1 and other DNA end-processing enzymes are inhibited through a physical structural “barrier” formed by Rad9 oligomers (53BP1 in mammals) bound near a DSB [10]. *RAD9* was originally identified as the first checkpoint gene in *S. cerevisiae* and recognized as an “adaptor” protein, linking the upstream kinase Mec1 to the activation of effector kinases Rad53 and Chk1. Rad9 is recruited to chromatin through three different pathways: i) the constitutive interaction with the histone H3 methylated at the K79 residue by Dot1 [20], [21], [22]; ii) the binding to the histone H2A phosphorylated at the S129 residue by Mec1 [23]; iii) the interaction with Dpb11 [24], [25]. In particular, phospho-H2A mediated Rad9 recruitment spreads many kilobases around a DNA lesion [26]; whereas Dpb11 appears to be more specific at the site of lesion, by binding to a damage-induced phosphorylation in the Ddc1 subunit of the 9-1-1 complex [25], [27], [28]. All of these three pathways cooperate for efficient checkpoint arrest and cell survival after genotoxic treatments throughout the cell cycle. Moreover, Rad9 contains motifs that are necessary for its oligomerization and DNA damage checkpoint signalling [24], [29], [30].

Notably, the Rad9-mediated inhibition of DSB resection is a regulatory function conserved throughout evolution. In fact, 53BP1 facilitates NHEJ at the expense of HR, protecting DNA ends from inappropriate 5' resection, in cooperation with the telomere binding protein RIF1 [31], [32], [33], [34], [35].

Here, we show that in the absence of Sae2, or in presence of mutations affecting Mre11 nuclease activity, Rad9 dimers and/or oligomers, recruited near a DSB mainly by Dpb11 interaction, inhibit the short-range DNA end processing, thereby preventing Mre11 removal from the lesion and limiting Rad52 recruitment by an Sgs1-dependent mechanism. As a consequence, DSB ends cannot be kept efficiently tethered to each other, and repair through an SSA process is prevented. We propose a novel molecular role of Rad9/53BP1 to protect genome integrity from extensive DNA degradation and rearrangements during DSB repair, also suggesting important implications for malignant transformation in mammalian cells.

## Results

### Deletion of *RAD9* gene rescues DSB repair defect in *sae2*Δ cells through an Sgs1-Dna2 dependent pathway

**Figure 1:**
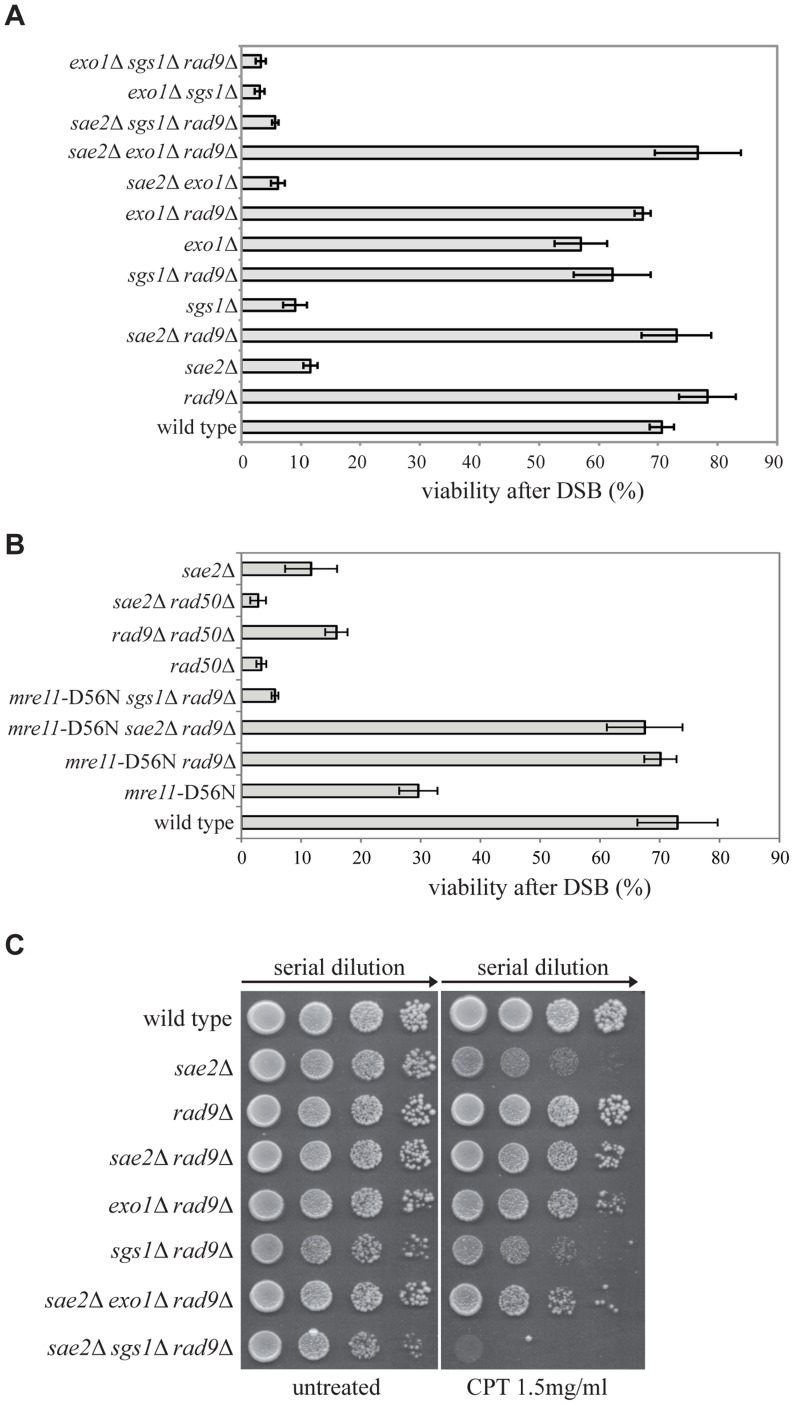
Deletion of *RAD9* rescues *sae2*Δ and *mre11*-D56N cell viability following DSBs through *SGS1*. (A–B) Viability of the wild type YMV80 strain and the indicated derivatives plated on YEP+gal. In the presence of galactose, one HO-cut is introduced at *leu2* locus (see a scheme in Fig. 2A). For each strain, the number of colonies grown after 3 days at 28°C in YEP+gal was normalized respect YEP+glu. Plotted values are the mean values ± SD from three independent experiments. (C) Exponentially growing cell cultures of the wild type YMV80 strain and the indicated derivatives were serially diluted (1∶10), and each dilution was spotted out into YPD and YPD+camptothecin plates. Plates were incubated 3 days at 28°C.

It is known that deletion of the *RAD9* gene in yeast leads to faster DSB resection and repair through an SSA process [36], [37]. To further understand the role of Rad9 in DSB processing and repair, we decided to combine the deletion of *RAD9* gene with mutations in genes encoding factors either involved in the short-range (*SAE2*), or the long-range (*EXO1*, *SGS1*) DSB resection [38]. We took advantage of the YMV80 background, in which the galactose-induced expression of the HO nuclease causes a single DSB at a specific site on chromosome III. Repair of this DSB occurs mainly through SSA between flanking homologous *leu2* repeats one of which is 25kb from the DSB [39]. We deleted *RAD9, EXO1*, *SGS1* and *SAE2* to obtain all viable single, double and triple mutant combinations. Although the *sae2*Δ *sgs1*Δ double mutant is a synthetic lethal combination [40], [41], *rad9*Δ interestingly suppresses *sae2*Δ *sgs1*Δ lethality (S1A Fig.). Therefore, it was possible to test the *sae2*Δ *sgs1*Δ *rad9*Δ triple mutant cells. After plating the cells in the presence of galactose to induce one DSB, we found that viability of the *sae2*Δ and *sgs1*Δ single mutant and *sgs1*Δ *exo1*Δ double mutant was severely reduced (Fig. 1A), as expected [6], [7], [42]. We also found that the deletion of *RAD9* gene effectively rescued the viability of the *sae2*Δ, *sgs1*Δ and *sae2*Δ *exo1*Δ mutant strains following one DSB (Fig. 1A). Interestingly, the viability of the *sae2*Δ *sgs1*Δ *rad9*Δ and *exo1*Δ *sgs1*Δ *rad9*Δ triple mutant cells was very low in the presence of one DSB. Moreover, the HO-induced lethality of the *sae2*Δ *sgs1*Δ *rad9*Δ mutant was not rescued by the expression of the Sgs1-K706A protein variant (S1B Fig.), whose helicase activity is severely reduced [43]. While the failure to repair the DSB in the *exo1*Δ *sgs1*Δ *rad9*Δ triple mutant was expected, since at least one of the Exo1 and Sgs1-dependent pathways is necessary to extensively resect a DSB, the result obtained with the *sae2*Δ *sgs1*Δ *rad9*Δ mutant was surprising. We therefore concluded that an Exo1-independent, Sgs1-dependent pathway is necessary for the viability of *sae2*Δ cells following a DSB in the absence of *RAD9*.

Since Sae2 stimulates the activity of the MRX complex in the first step of the DSB end processing [44], we considered the possibility that *RAD9* deletion may also rescue an Mre11 nuclease defective mutant or the *rad50*Δ mutant, in which the MRX complex is disassembled. Interestingly, we found that *rad9*Δ suppresses the nuclease-defective *mre11*-D56N mutant [45], through an *SGS1*-dependent pathway, while it does not rescue *rad50*Δ mutant, as expected [36] (Fig. 1B). These results suggest that the nuclease activity of the MRX complex is dispensable for the DSB repair in *rad9*Δ cells; however, the MRX complex must be physically present, likely playing an essential structural role. Indeed, *rad50*Δ mutation does not rescue *sae2*Δ cell viability following a DSB (Fig. 1B). Of note, deletion of *RAD9* also suppresses the double mutant *mre11*-D56N *sae2*Δ, further indicating that Mre11 and Sae2 work together in the same pathway (Fig. 1B).

Importantly, the deletion of *RAD9* rescues *sae2*Δ cell viability through an *EXO1*-independent, *SGS1*-dependent pathway also in presence of camptothecin (Fig. 1C), a topoisomerase-aborting agent that causes formation of end-blocked DSBs [46].

**Figure 2:**
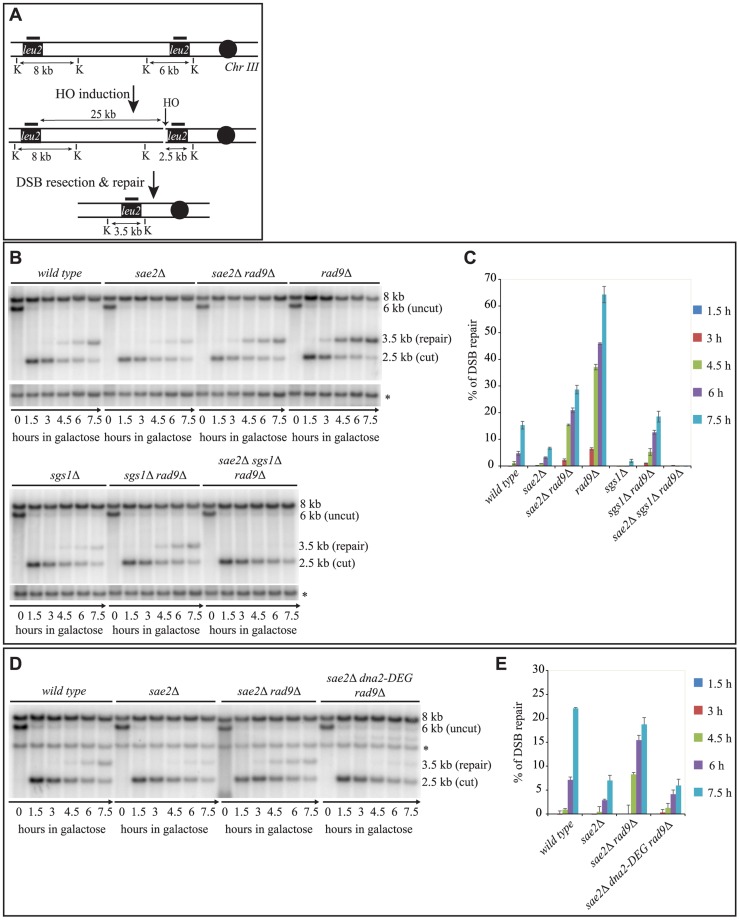
Deletion of *RAD9* rescues DSB repair defects of *sae2*Δ cells through *SGS1* and *DNA2*. (A) Map of the YMV80 Chr III region, containing the HO-cut site. The indicated vertical bars show KpnI restriction sites. The short thick lines indicate the position where the probe hybridizes. After the HO mediated cleavage, DNA ends are resected. Once the indicated *leu2* cassettes have been exposed as ssDNA, repair through SSA can occur and be monitored by the appearance of an SSA product fragment by Southern blot. (B and D) Exponentially growing YEP+raf cell cultures of the wild type YMV80 strain and the indicated derivatives were synchronized and kept blocked in G2/M phase with nocodazole treatment; galactose was added at time zero to induce HO-cut. *KpnI*-digested DNA was analysed by Southern blotting with a *LEU2* probe. An *ATG5* (uncut locus on chromosome XVI) probe was also used to normalize the signals. In (D) *LEU2* and *ATG5* probes were added contemporarily to the filter. (C and E) Densitometric analysis of the product band signals of the experiments shown in (B) and (D). The intensity of each band was normalized respect to unprocessed *ATG5* locus (*).

To further investigate the findings shown in Fig. 1A at the molecular level, we tested the kinetics of DSB repair by Southern blotting in cells blocked in G2/M cell cycle phase by nocodazole. In agreement with the cell lethality reported in Fig. 1A, we found that the efficiency of the DSB repair is reduced in both the *sae2*Δ and *sgs1*Δ single mutants, as previously described [6], [7], [42], and it is severely compromised in *sae2*Δ *sgs1*Δ *rad9*Δ (Figs. 2B and 2C). On the contrary, DSB repair is accelerated and very efficient in the *rad9*Δ, *sae2*Δ *rad9*Δ and *sgs1*Δ *rad9*Δ mutants (Figs. 2B and 2C). These results indicate that, in the absence of Rad9, an Sgs1-dependent mechanism is necessary to efficiently repair a DSB in *sae2*Δ cells.

To test if Sgs1 cooperates with Dna2 to repair a DSB in *sae2*Δ *rad9*Δ mutant cells, we took advantage of an auxin-based degradable Dna2 protein variant (Dna2-DEG). This is a common genetic strategy to induce the degradation of a protein by the addition of auxin compound to the cell culture medium [47], and it is particularly useful in the case of an essential gene, such as *DNA2*. By Southern blotting analysis, we found that the *sae2*Δ *rad9*Δ double mutant cells do not repair a DSB in the absence of Dna2 (Fig. 2D and 2E). Therefore, taking all the data in Fig. 2 together, we concluded that the deletion of *RAD9* rescues *sae2*Δ cells through a DSB resection mechanism mediated by the Sgs1-Dna2 pathway.

In addition, we ruled out the possibility that in the absence of Rad9, the DSB can be repaired more efficiently through a strand invasion-based mechanism (such as a break-induced replication process [48]). In fact, we observed faster DSB repair and high viability when we analysed the *sae2*Δ *rad9*Δ *rad51*Δ triple mutant, in which break-induced replication is impaired, but SSA is not inhibited (S2 Fig.).

### Rad9 limits an Sgs1- and Sae2- dependent initial step of DSB processing

**Figure 3:**
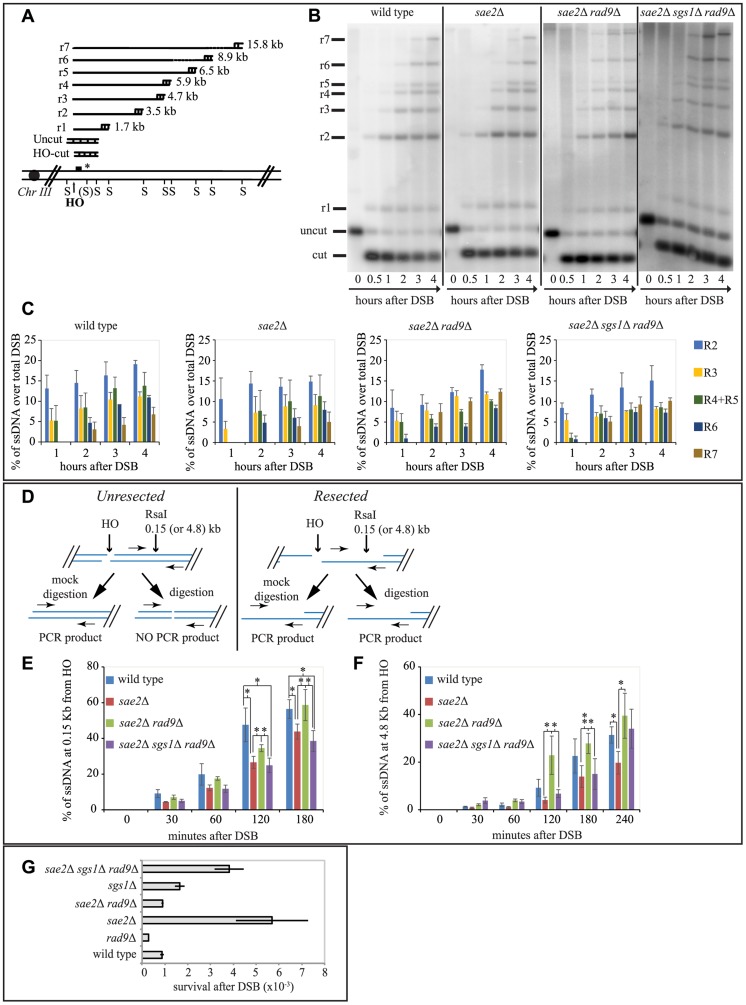
Rad9 limits an Sgs1- and Sae2- dependent initial step of DSB resection. (A) Scheme of the *MAT* locus. The figure shows the positions of the HO-cut site, and the probe used in experiments shown in (B and C) and in S3 and S4 Figs. (B, C) Exponentially growing YEP+raf cell cultures of the wild type JKM139 strain and the indicated derivatives, carrying a unique HO cut site at *MAT* locus and expressing the HO nuclease under GAL1 promoter, were synchronized and kept in G2/M phases by nocodazole treatment. Galactose was added at time 0 to induce HO. SspI-digested genomic DNA, extracted from samples taken at the indicated times, was analysed by Southern blotting to test 3′ filament formation. (C) The mean values ± SEM corresponding to the resection products of two independent experiments were determined by densitometry. (D) Schematic representation of the quantitative PCR method used to monitor HO-induced DSB resection. (E–F) Plots showing the ratio of resected DNA among HO cut DNAs at each time points by qPCR analysis. The mean values from three independent experiments are shown with SEM. Significance was calculated by one-tailed paired Student's *t* test (* for P<0.05; ** for P<0.01; where not indicated, the P value was higher than 0.05) (G) JKM139 derivatives were nocodazole-arrested in G2/M and 2% galactose was added to induce HO cut. After 2 hours of HO induction, cells were plated on YEP+raf and YEP+raf+gal, and incubated at 28°C for three days. Viability results were obtained from the ratio between number of colonies on YEP+raf+gal and YEP+raf. The mean values from three independent experiments are shown with SD.

A critical step to repair a DSB through SSA is 5′ to 3′ resection of the DSB end. Therefore, based on our results in Figs. 1 and 2, we hypothesized that in *sae2*Δ *sgs1*Δ *rad9*Δ triple mutant DSB resection may be affected, as it was shown in the *sae2*Δ single mutant [6], [7], [42], while it should be faster in *sae2*Δ *rad9*Δ double mutant. To test the kinetics of DSB processing we used JKM139 background derivatives, where prolonged expression of HO causes an irreparable DSB at *MAT* locus, because of the absence of *HML* and *HMR* homologous cassettes. Therefore, the analysis of the formation of the 3′ single-stranded (ss) DNA is not biased by a repair process [49]. Using Southern blotting of denatured DNA after restriction enzyme digestion [50], we tested the formation of the 3′ ssDNA filament (as depicted in Fig. 3A), after the induction of one DSB in each sister chromatid, in G2/M-blocked cells.

As expected, we found that the formation of a long 3′ ssDNA tail is slightly delayed in the absence of *SAE2*, *EXO1* and *SGS1* genes, and it is severely compromised in the *exo1*Δ *sgs1*Δ double mutant [6], [7], [51]. Interestingly, we found more extensive 3′ ssDNA in the absence of Rad9 in all the mutants tested, except the *exo1*Δ *sgs1*Δ *rad9*Δ triple mutant (Figs. 3B, 3C and S3). These results support the model that both the Exo1 and the Sgs1-dependent pathways cooperate to resect a DSB, and rule out the hypothesis that additional nuclease(s) may take over to process a DSB in the absence of Rad9. However, we noticed that in the *sae2*Δ *sgs1*Δ *rad9*Δ triple mutant strain the appearance of ssDNA is slightly delayed compared to wild type and *sae2*Δ *rad9*Δ strains (Figs. 3B and 3C). This result may suggest that the initiation of DSB resection is affected in *sae2*Δ *sgs1*Δ *rad9*Δ cells.

To test more precisely DNA processing near a DSB we employed a quantitative PCR-based method [52]. In particular, by this procedure we determined if the *Rsa*I restriction enzyme can cut the DNA at a specific site 150 bp from the HO-cut site, thus indicating whether DSB resection has already passed beyond this site, since, as resection proceeds, the *Rsa*I site becomes single stranded and resistant to digestion, which results in a PCR fragment amplification (see scheme in Fig. 3D). Thus, the rate of PCR fragment amplification, normalized to the efficiency of HO cutting, corresponds to the rate of resection [52]. We also tested with the same procedure another *Rsa*I site 4800 bp from the HO cut site, as a control. Interestingly, we noticed a higher amount of un-resected DNA at 150 bp proximal the DSB site, between 60 and 180 minutes after the cut in nocodazole blocked *sae2*Δ and *sae2*Δ *sgs1*Δ *rad9*Δ triple mutant cells with respect to the wild type and *sae2*Δ *rad9*Δ mutant (Fig. 3E). However, at later time points resection has efficiently passed beyond the *Rsa*I site 4800 bp far from the HO cut site (Fig. 3F), not only in the wild type and *sae2*Δ *rad9*Δ cells, but also in the *sae2*Δ *sgs1*Δ *rad9*Δ triple mutant cells, according to the visualization of the 3′ ssDNA formation by denaturing Southern blotting (Figs. 3B and 3C).

These studies revealed one striking unexpected result: although *sae2*Δ *sgs1*Δ *rad9*Δ triple mutant cells resect a DSB and expose an extended 3′ ssDNA (Figs. 3B, 3E and 3F), they are severely compromised in DSB repair through SSA (Figs. 2B and 2C), suggesting that the long-range resection is not the limiting step to repair a DSB in these cells, rather the defect is different from simply creating enough ssDNA to allow SSA to take place. Therefore, we hypothesize that an Sgs1-dependent mechanism contributes to efficiently initiate DSB processing in the absence of both Rad9 and Sae2, and the kinetics of the initial step of resection would become somehow critical to complete the subsequent steps of the SSA repair.

We then investigated whether the faster DSB end processing that we observed in *sae2*Δ *rad9*Δ cells would be associated with reduced NHEJ events, which are significantly elevated in the *sae2*Δ cells [53]. To this aim, we treated cells of JKM139 strains with nocodazole to block cell cycle in G2/M phase and we added galactose to induce one persistent DSB in each sister chromatid. Cells were kept in nocodazole for 2 hours to avoid potential interference caused by cell cycle transition, before plating in the presence of galactose. In this condition, the continued expression of HO leads to a recurrent cut of the *MAT* locus and precludes precise religation, until the sequence of the HO site is corrupted by deletion/addition of few bases and the ends are joined by imprecise NHEJ [54]. This is a relatively inefficient process in yeast, with a frequency of about 1-3×10^−3^ in wild type cells [54]. We found that the frequency of imprecise NHEJ events is increased in *sae2*Δ cells, in agreement with previous finding [53], while it is slightly reduced in the absence of Rad9. Interestingly, deletion of *RAD9* reduces NHEJ events to wild type value in *sae2*Δ cells (Fig. 3G).

These results suggest that Rad9 plays a critical role to balance NHEJ and HR events in G2/M phase, likely acting at an early step of DSB processing, leading to increased NHEJ events in the absence of Sae2.

### Rad9 limits Mre11 removal from a DSB, affecting Rad52 binding and DSB end-tethering in *sae2*Δ cells

**Figure 4:**
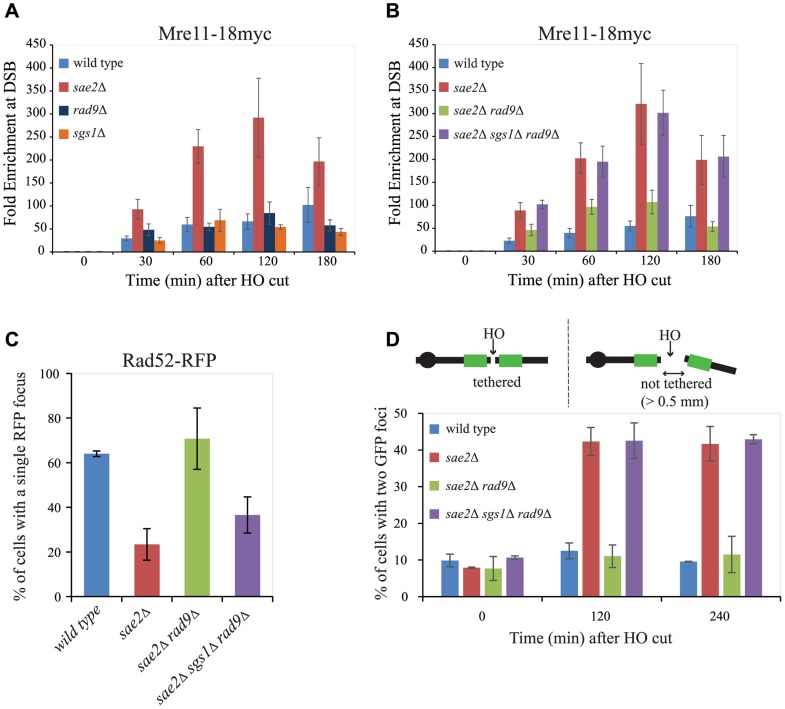
Rad9 limits Mre11 removal from a DSB, affecting Rad52 binding and DSB ends tethering in *sae2*Δ cells. (A, B) Cells of the wild type JKM139 strain and the indicated derivatives, expressing a Mre11–18Myc fusion protein, were grown in YEP+raf and synchronized in G2/M phases by nocodazole treatment. Galactose was added at time 0 to induce HO. Relative fold enrichment of Mre11–18Myc at 0.1 kb from the HO cleavage site was evaluated after ChIP with anti-Myc antibodies and qPCR analysis. Plotted values are the mean values ± SEM from three independent experiments. (C) Cells of the wild type JKM139 strain and the indicated derivatives, expressing a Rad52-RFP fusion protein, were grown in YEP+raf and synchronized in G2/M phases by nocodazole treatment. Galactose was added at time 0 to induce HO. After 6 hours from DSB, cells were imaged under live cell conditions for Rad52-RFP focus formation. Approximately 100 cells per experiment were analyzed and the percentage of cells displaying a detectable Rad52-RFP focus was quantitated. Error bars reflect ranges from two independent experiments. (D) Cells of the wild type yJK40.6 strain and the indicated derivatives, expressing a LacI-GFP and carrying two *LacO* arrays (green boxes) at 50 kb on either side of one HO cut site on chromosome VII (see a scheme above the graph in Fig. 4D and text for details), were grown in YEP+raf and blocked in G2/M phases by nocodazole treatment. Galactose was added at time 0 to induce HO. Cell samples taken at the indicated times after HO induction were analysed with a fluorescence microscope to determine the percentage of cells in each sample that contained two LacI-GFP foci separated by>0.5 µm. The separation distance between foci was measured for 200 cells/sample.

The delay in DSB resection in *sae2*Δ cells has been correlated with a prolonged Mre11 binding at the DSB site [42], [55]. More recently, it was also shown that an Sgs1-dependent process can contribute to remove Mre11 from a DSB in *sae2*Δ cells, promoting DSB resection and repair through homologous recombination [56]. Therefore, we decided to investigate Mre11 binding near a DSB by a chromatin immunoprecipitation-after-crosslinking-protocol (ChIP), followed by quantitative PCR (qPCR), with primers specific for the DSB site. Contrary to wild type, *rad9*Δ or *sgs1*Δ cells, we found greater and persistent levels of Mre11 bound near DSB ends in *sae2*Δ cells (Fig. 4A), supporting previous analysis of the Mre11 foci by microscopy [51], [56], and by ChIP [55]. Importantly, we found a decrease in fold enrichment of Mre11 binding to the DSB site in *sae2*Δ *rad9*Δ cells, but not in the *sae2*Δ *sgs1*Δ *rad9*Δ triple mutant cells (Fig. 4B). These results suggest that the deletion of *RAD9* gene promotes an Sgs1-dependent process to remove Mre11 from DSB ends in the absence of Sae2, supporting and expanding recent findings [56], and it may explain the high efficiency of SSA repair and viability of the *sae2*Δ *rad9*Δ that we showed in Figs. 1 and 2. Moreover, the prolonged binding of Mre11 near the DSB further supports previous results in Fig. 3, showing that short-range resection in the *sae2*Δ and *sae2*Δ *sgs1*Δ *rad9*Δ triple mutant cells is delayed.

Since it is known that Mre11 persistence at a DSB limits the recruitment of Rad52 [4], [57], which is necessary to establish DNA end-tethering and HR pathways [58], [59], we investigated by immunofluorescence Rad52 loading onto one DSB in all the mutants described. We found that deletion of *RAD9* totally restores Rad52 binding in *sae2*Δ cells through an Sgs1-dependent mechanism (Fig. 4C). These results correlate with the analysis of Mre11 binding in these mutants (Fig. 4B), and suggest that the limiting step to efficiently complete an SSA process in nocodazole-blocked *sae2*Δ and *sae2*Δ *sgs1*Δ *rad9*Δ cells is not the delay in DSB resection *per se* (Figs. 3B and 3C), but rather the reduced binding of Rad52.

Rad52 is a critical factor to maintain DSB ends tethered to each other, which was suggested to be a relevant event in HR [42], [58], [59], [60], [61]. As we showed that the deletion of *RAD9* allows Rad52 binding in *sae2*Δ cells (Fig. 4C), we investigated whether it may also contribute to rescue DSB end-tethering defect in these cells. To this end, we took advantage of a specific yeast background in which the DNA proximal to the irreparable HO break could be visualized by binding of a LacI-GFP (green fluorescent protein) fusion protein to multiple repeats of the LacI repressor binding site, *LacO*. These arrays are integrated at a distance of 50 kb on either side of the HO cleavage site on chromosome VII [58]. Cultures of the original wild type and isogenic *sae2*Δ, *sae2*Δ *rad9*Δ and *sae2*Δ *sgs1*Δ *rad9*Δ derivative strains were arrested in mitosis and kept blocked by nocodazole treatment during break induction by galactose addition. After 2 hours to ensure HO cut formation, we observed two LacI-GFP spots in only 12.5%±2.1% of the wild type cells, and 11.0%±3.1% in *sae2*Δ *rad9*Δ mutant cells, thus indicating their ability to hold the broken DNA ends together. In contrast, 42.3%±3.8% of *sae2*Δ and 42.5%±4.8% of *sae2*Δ *sgs1*Δ *rad9*Δ cells showed two LacI-GFP spots, indicating a failure in DSB end-tethering (Fig. 4D, and see also [42], [62]).

Therefore, we conclude that the deletion of *RAD9* rescues both the Rad52 binding and DSB end-tethering in *sae2*Δ cells, contributing to efficiently repair a DSB through an SSA process that requires the resection of 25 kb of DNA between the repeats (Fig. 2A).

### Rad9 oligomers limit *sae2*Δ cells viability following a DSB mainly through the interaction with Dpb11

**Figure 5:**
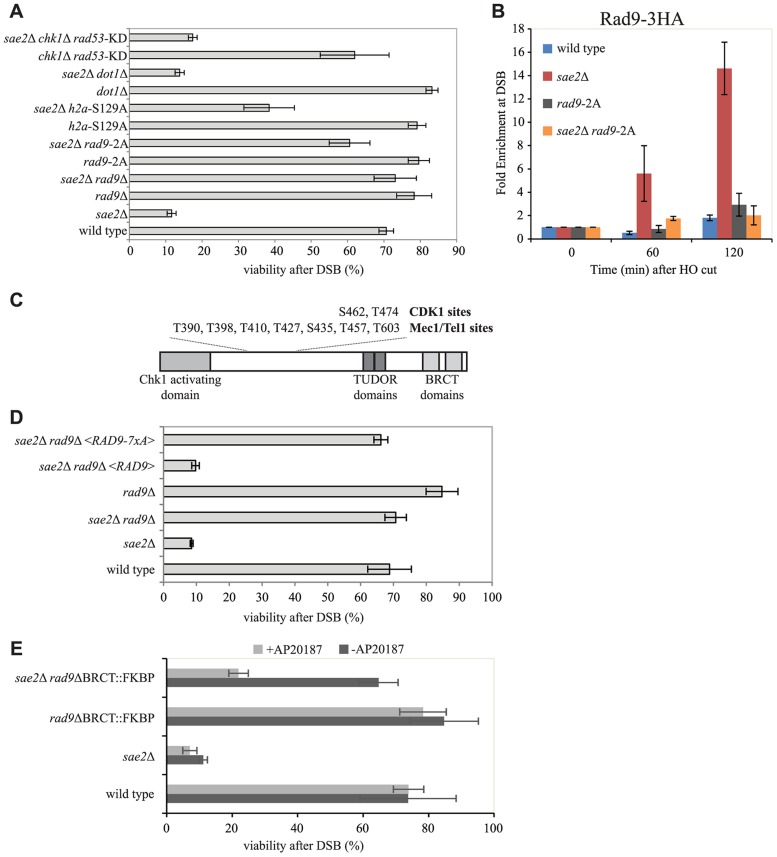
Rad9 oligomers affect cell viability following a DSB, in the absence of Sae2, mainly through the interaction with Dpb11. (A and D) Viability of the wild type YMV80 strain and the indicated derivatives, plated on YEP+raf+gal. For each strain, the number of colonies grown after 3 days at 28°C in YEP+raf+gal was normalized respect YEP+raf. Plotted values are the mean values ± SD from three independent experiments. (B) Cells of the wild type JKM139 strain and the indicated derivatives, expressing a Rad9-3HA fusion protein, were grown in YEP+raf and synchronized in G2/M phases by nocodazole treatment. Galactose was added at time 0 to induce HO. Relative fold enrichment of Rad9-3HA at 0.1 kb from the HO cleavage site was evaluated after ChIP with anti-HA antibodies and qPCR analysis. Plotted values are the mean values ± SEM from three independent experiments. (C) Schematic representation of Rad9 functional domains and sites phosphorylated by CDK1, Mec1 and Tel1. (E) Exponentially growing cell cultures of the wild type YMV80 strain and the indicated derivatives were incubated for 2 hours with or without the dimerization-inducing molecule AP20187, before plating in YEP+Raf or YEP+Raf+Gal, with/without AP20187. For each strain, the number of colonies grown after 3 days at 28°C in YEP+raf+gal was normalized with respect to YEP+raf. Plotted values are the mean values ± SD from three independent experiments. Expression level of Rad9-2A, Rad9-7xA and Rad9-ΔBRCT-FKBP protein variants, described in this Figure, were determined by western blotting in S6 Fig.

It was previously suggested that Rad9 limits DSB resection acting as a physical barrier toward the actions of nucleases, through a function distinct from its role in DNA damage checkpoint signalling [10]. Therefore, we sought to address if a checkpoint-independent function of Rad9 was involved to limit *sae2*Δ cells viability following one DSB. To this aim, we tested the *chk1*Δ *rad53*-K227A double mutant in the YMV80 background, in which the Rad53 kinase activity is dead and both the two checkpoint-signaling pathways acting downstream Rad9 are abrogated. By plating the cells in the presence of galactose to induce one HO cut, we found that the viability of the *sae2*Δ *chk1*Δ *rad53*-K227A triple mutant cells is reduced, similarly to *sae2*Δ cells (Fig. 5A). This result indicates that signaling through Rad53 and/or Chk1 is not involved into the mechanism by which Rad9 limits SSA repair in *sae2*Δ cells.

In order to further understand how Rad9 inhibits SSA repair in *sae2*Δ cells, we then investigated specific mutations that affect Rad9 binding to a DSB. It is known that Rad9 constitutively binds chromatin through the interaction between its TUDOR domain and the histone H3 methylated at the K79 by Dot1 [20], [21], [22]. In addition, Rad9 binds chromatin around a DSB site through the interaction of its BRCT domain with the histone H2A phosphorylated at the S129 (γ-H2AX) by upstream kinase Mec1 and Tel1 [23]. Further, Rad9 is recruited near a DNA lesion through the interaction with Dpb11 protein. In particular, Dpb11 binds the CDK1-dependent phosphorylated S462 and T474 Rad9 residues, reinforcing the Rad9 binding to damaged DNA and promoting Rad9 phosphorylation by Mec1 [25].

To test the contribution of the different pathways that mediate Rad9 binding to chromatin, we analysed the viability in the presence of HO-induced DSB of specific mutations that abrogate each of them in the YMV80 background. The deletion of *DOT1* gene eliminates the H3K79 methyl transferase Dot1 protein, and greatly reduces the constitutive binding of Rad9 to chromatin [21], [24]. As expected [36], deletion of *DOT1* leads to a faster long-range DSB resection in *sae2*Δ cells (S4A and S4B Figs.). However, by the qPCR-based method, we found that the initial short-range resection is still delayed in these double mutant cells (S4C Fig.), suggesting that the Dot1-dependent resection barrier may have a role only at distal region from the cut site. Indeed, by plating the YMV80 derivative cells in the presence of galactose to induce one DSB, we found that deletion of *DOT1* gene does not rescue *sae2*Δ lethality (Fig. 5A). Further, we deleted *SAE2* gene in a strain that expresses the H2A-S129A histone variant, which is not phosphorylatable by Mec1 and Tel1 kinases and leads to a faster DSB resection [63]. We also deleted *SAE2* gene in a strain that expresses the Rad9-S462A-T474A (hereafter we refer to *rad9*-S462A-T474A as *rad9*-2A) protein variant, which does not interact with Dpb11 [25]. Interestingly, both the failure to phosphorylate the H2A-S129 site and the *rad9*-2A mutation increase the viability of *sae2*Δ cells after one DSB, with the major contribution done by the mutation that abrogates the Rad9-Dpb11 interaction (Fig. 5A). Taking all these genetic results together, we concluded that the recruitment of Rad9 near the DSB site, mediated by its interaction with Dpb11 and partially with γ-H2AX, limits *sae2*Δ cells viability when a DSB must be repaired by SSA.

Consistently with our genetic evidence, we found an increased binding of Rad9 close to an irreparable DSB in *sae2*Δ cells by ChIP analysis (Fig. 5B), which correlates with the increased binding of Mre11 (Figs. 4A and 4B). Of note, the Rad9-2A protein variant does not bind near a break (Fig. 5B), supporting the viability data of the *sae2*Δ *rad9*-2A double mutant cells following one DSB (Fig. 5A). Moreover, Rad9 binding close to the break is only partially dependent on γ-H2AX and not by Dot1 (S5 Fig.), in agreement with cell viability of the *sae2*Δ h2a-S129A and *sae2*Δ *dot1*Δ double mutants (Fig. 5A).

Then we tested if the capability of Rad9 to form oligomers at the DNA damage site [29], [30], [64] was involved in inhibiting *sae2*Δ cells viability following a DSB. To this aim, we introduced a plasmid vector that expresses either the *rad9*-7xA allele or the *RAD9* gene as a control, by transformation into *rad9*Δ and *sae2*Δ *rad9*Δ YMV80 derivatives. The Rad9-7xA protein variant cannot be phosphorylated at critical sites by upstream Mec1 and Tel1 kinases (see also Fig. 5C), and is unable to oligomerize [29], [64]. After plating cells in the presence of galactose to induce one DSB, we found that the expression of the Rad9-7xA protein variant rescues the lethality of *sae2*Δ cells, contrary to the wild type Rad9 (Fig. 5D). This result suggests that the oligomerization of Rad9 molecules is implicated in limiting SSA repair in *sae2*Δ cells. To further support this conclusion, we took advantage of the *rad9*-ΔBRCT-FKBP chimeric allele, which leads to the production of a truncated variant of Rad9 protein, in which the C-terminal BRCT domains are replaced with a FKBP tag [24]. It was shown that the Rad9-ΔBRCT-FKBP protein variant, which cannot form oligomers due to the absence of the BRCT domains, can dimerize in the presence of the small inducing molecule AP20187, binds chromatin and partially transduces the checkpoint signal (S6B Fig. and see also [24]). Consistent with our hypothesis, we found that the *rad9*-ΔBRCT-FKBP mutation does not rescue *sae2*Δ lethality in the presence of AP20187, while the viability in the *sae2*Δ *rad9*-ΔBRCT-FKBP double mutant cells is almost identical to the wild type value (Fig. 5E), further suggesting that the dimerization/oligomerization of Rad9 affects SSA repair.

## Discussion

It is now clear that DSB processing is a finely regulated process, which acts at the crossroad between HR and NHEJ recombination pathways. Indeed, as soon as a DSB is resected, homologous recombination pathways can be used to repair the break in lieu of NHEJ, with important implications for chromosome rearrangements and genome integrity.

Similarly to what seen in higher eukaryotes, three distinct nucleases cooperate to resect a DSB in *S. cerevisiae*. According to a model recently proposed for meiotic DSBs [65], Mre11, activated by Sae2 [44], introduces a nick near a DSB, triggering a bidirectional nucleolytic degradation of the 5′ strand: Exo1 and Dna2-Sgs1 resect the DNA in the 5′-to-3′ direction from the nick, while the Mre11 complex resects the DNA in the 3′-to-5′ direction toward the DSB ends. In G2/M blocked cells, it appears that the Exo1 and Dna2-Sgs1 pathways cannot actively resect a DSB starting from its ends, which are occupied by Ku70-Ku80 complex [1]. Indeed, it was suggested that the Mre11 activity might contribute to the removal of Ku complex, clearing the ends [2], [3], [11], [65], [66]. Importantly, in the absence of a functional Sae2, the Mre11-dependent DSB processing is compromised, and Ku-dependent NHEJ events and translocations increased [62]. In addition, Mre11 and Rad52 binding are, respectively, increased and reduced in *sae2*Δ cells (Fig. 4, and see [4], [57]), which are severely defective in repairing a DSB through SSA (Fig. 2, and see also [6], [42]). Moreover, *sae2*Δ cells cannot keep the DSB ends tethered, which was shown to be relevant for DSB repair (Fig. 4, and see [42], [58], [60]). Here, we show that the deletion of the *RAD9* gene suppresses all these phenotypes of *sae2*Δ cells. Indeed, we found that deletion of *RAD9* leads to a faster 5′–3′ resection both through the Exo1 and Dna2-Sgs1 pathways, but the Dna2-Sgs1 pathway becomes essential, in the absence of Sae2, to efficiently initiate DSB processing and repair through an SSA process that requires 25 kb DNA resection (Figs. 2 and 3). We also found elevated levels of Mre11 bound near an HO-induced break both in *sae2*Δ and *sae2*Δ *sgs1*Δ *rad9*Δ mutants, accordingly with a defect in Rad52 binding and DNA end-tethering (Fig. 4). The requirement of DSB end-tethering for SSA repair has never been explored before, however it is relevant to underline that Rad52 is important for end-tethering [58], and also our results indicate that a defect in end-tethering is linked with a failure to accomplish SSA repair. Further investigation will be required to fully understand the interplay between SSA and end-tethering. Interestingly, recent findings underlined a role of exonuclease processing of a DSB in maintaining broken chromosome ends in close proximity [61].

Taken all these findings together, we suggest that the prolonged binding of Mre11 near the break site may represent the critical barrier to efficiently initiate DSB resection, load Rad52 and establish end-tethering in the absence of Sae2, and it can be by-passed by a resection-based mechanism mediated by Sgs1-Dna2 in the absence of Rad9.

A similar role to remove Mre11 from a DSB site in *sae2*Δ cells was recently shown for Sgs1, in the absence of Ku70-Ku80 complex [56]. Indeed, deletion of *KU70* suppresses *sae2*Δ cells sensitivity to low doses of CPT and other DSB inducing agents [1], [3]. Surprisingly, we did not see a rescue of *sae2*Δ cells lethality by deleting *KU70* after a DSB that can be repaired through an SSA process between two homologous *leu2* repeats 25kb far from each other, although deletion of *RAD9* suppresses the *sae2*Δ *ku70*Δ double mutant (S7 Fig.). One possibility is that Rad9, bound near a DSB site, may limit the Sgs1-Dna2 activity starting from the break ends, leading to prolonged Mre11 binding. This might occur in cooperation with Ku complex, bound to the DSB ends, or rather it might represent a second distinct mechanism to limit DSB ends resection and DNA end-tethering. Alternatively, or in addition, Ku and Rad9 may limit DSB processing in different cell cycle phases. Indeed, the Ku complex acts on a DSB mainly in G1, while Rad9 acts predominantly in G2/M phase [36], [67], [68].

Genetic and biochemical evidence in Fig. 5 suggest that Rad9 protein dimerization and/or oligomerization, together with Rad9 interactions with Dpb11 and partially with γ-H2AX, are important to limit short-range resection and repair in *sae2*Δ cells. Indeed, Dpb11 is recruited on to the DNA lesion through the interaction with the 9-1-1 complex [28], and both the 9-1-1 complex and Dpb11 are recruited rapidly near a DSB site [69], likely at the ssDNA-dsDNA junction [70]. It is possible that the interactions with γ-H2AX, as well as with the histone H3 methylated at Lys79 by Dot1, become more important to recruit Rad9 in a distal region from the DSB site, contributing to slow down the long-range resection, which is not the limiting step in *sae2*Δ cells. This hypothesis is supported by the fact that DNA damage sensitivity of *fun30*Δ cells, that resect slower a DSB because of their inefficient Rad9 removal from chromatin flanking a DSB [37], is partially rescued in the absence of γ-H2AX or Dot1 [37], [63]. Of importance, deletion of *DOT1* gene does not rescue *sae2*Δ cells (Fig. 5A). Notably, although Rad9 binding close to the break is not particularly elevated in wild type cells, it is enriched in *sae2*Δ cells (Fig. 5C). Consistent with our genetic evidence, Rad9 binding close to DNA ends depends on Dpb11, partially on the histone γ-H2AX, but not on the histone H3 methylated at Lys79 by Dot1 (Figs. 5B and S5). Possibly, these data are in agreement with the low amount of modified histones detected in chromatin within 1–2 kb of the break [22], [26], [71], [72], [73].

**Figure 6:**
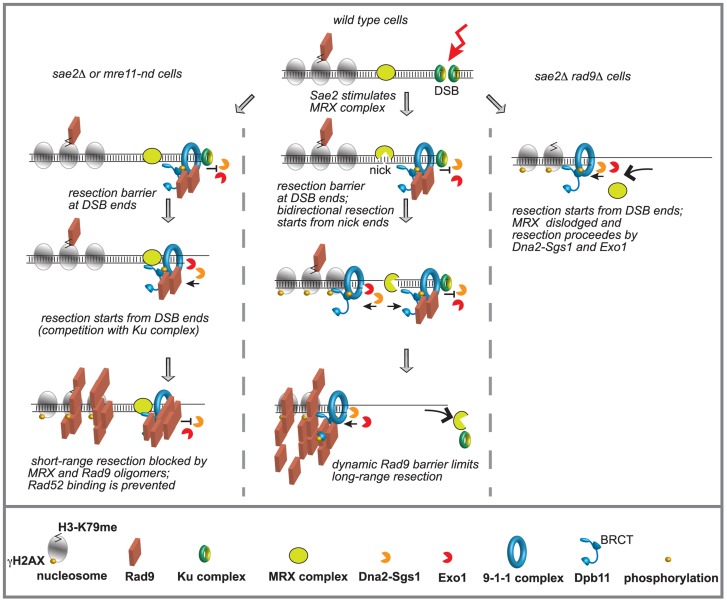
Model to explain the interplay between Mre11 complex and Rad9 at a DSB in G2/M phase. Ku and Mre11 complexes, together with Rad9, are recruited soon after a DSB formation and limit the action of Exo1 and Dna2-Sgs1 pathways. The order of appearance of the various factors was based on both literature and our results. See details in the text.

Overall, our genetic and molecular results suggest a model shown in Fig. 6, in which Rad9, in addition to its known role in inhibiting long-range resection, may affect the initial short-range processing of an HO-induced DSB. In fact, Rad9, once recruited close to a DSB end in G2 phase mainly through the interaction with Dpb11, limits the Sgs1 dependent resection starting from DNA ends, whenever Mre11 is blocked near the DNA ends. In the future it will be interesting to investigate whether Rad9 plays a similar role in limiting rapid and coincident resection of dirty radiation-induced DSBs, in cells lacking Sae2 and/or Mre11 [74].

We believe that our findings might have important implications for understanding how the genome stability is preserved, especially in higher eukaryotes, whose genomes are enriched of repeats and SSA events can be particularly frequent. In fact, it becomes clear that too-efficient DSB resection can lead to an excessive initiation of homologous recombination and accumulation of toxic DNA intermediates and rearrangements between repeats [16]. Moreover, DSB resection may lead to highly error-prone alternative ends joining (A-EJ) and MMEJ events [14], [16]. In this view, our results in yeast might help to understand recent finding in human cells at the molecular level, showing a role for 53BP1 in protecting from BLM and CtIP-Mre11 dependent A-EJ events and genome rearrangements [75].

Furthermore, our findings suggest that the functional interplay between 53BP1/Rad9 and Mre11 may also have a physiological relevance to protect from error-prone imprecise NHEJ events in genomic regions containing no repeats. It is also worth mentioning that the inactivation of 53BP1 was shown to potentiate homologous recombination and increase DNA damage tolerance of cancer-prone BRCA1 -/- cells [32], [76], [77], [78], with severe implications for therapeutic treatments.

In conclusion, we show novel insights on the structural barrier induced by Rad9, together with Dpb11 and γ-H2AX, to limit DSB processing and repair. The Sgs1-Dna2 pathway becomes essential to efficiently remove hypo-active Mre11 from a DSB site, in the absence of Sae2 and Rad9, triggering DSB resection and repair. The efficient removal of Mre11 from the DSB site is essential not only to switch to the more processive long-range resection, but also to allow an efficient recruitment of the recombination factor Rad52. This allows the maintenance of DSB end-tethering, which is an important prerequisite to complete repair, especially for those lesions that require extensive resection. These events increase in the absence of Rad9 and might contribute to accumulation of toxic HR events, leading to genome rearrangements and genetic instability.

## Materials and Methods

### Yeast strains, media and growth conditions

All the strains listed in S1 Table are derivative of JKM139, YMV80 and yJK40.6. To construct strains standard genetic procedures of transformation and tetrad analysis were followed. Deletions and tag fusions were generated by the one-step PCR system [79]. For the indicated experiments, cells were grown in YP medium enriched with 2% glucose (YEP+glu), raffinose 3% (YEP+raf) or raffinose 3% and galactose 2% (YEP+raf+gal). All the synchronization experiments were performed at 28°C.

### Measurement of DSB resection at MAT locus

DSB end resection in JKM139 derivative strains was analyzed on alkaline agarose gels using a single-stranded RNA probe as described previously [36], [50].

### SDS-PAGE and western blot

TCA protein extract was prepared [80] and separated by SDS-PAGE. Western blotting was performed with anti-Rad53 (EL7), anti-HA (12CA5), anti-Rad9 (generously provided by N. F. Lowndes), and anti-actin using standard techniques.

### Analysis of SSA repair

Repair of an HO-induced DSB in YMV80 background was analyzed by a Southern blotting procedure described previously [39].

### Cell viability assay

YMV80 derivative strains were inoculated in YEP+raf, grown O/N at 28°C. The following day, cells were normalized and plated on YEP+raf and YEP+raf+gal. Plates were incubated at 28°C for three days. Viability results were obtained from the ratio between number of colonies on YEP+raf+gal and YEP+raf. Standard deviation was calculated on three independent experiments.

### Non homologous end joining assay

JKM139 derivative strains were inoculated in YEP+raf, grown O/N at 28°C. The following day, after cell cycle block in G2/M by nocodazole, 2% galactose was added to one part of the culture to induce HO cut. After 2 hours of HO induction, cells were normalized and plated on YEP+raf and YEP+raf+gal. Plates were incubated at 28°C for three days. Viability results were obtained from the ratio between number of colonies on YEP+raf+gal and YEP+raf. Standard deviation was calculated on three independent experiments.

### ChIP analysis

ChIP analysis was performed as described previously [69]. Input and immunoprecipitated DNA were analysed by quantitative PCR using a Biorad MyIQ2 system or a Biorad CFX connect. The oligonucleotides used are listed in S2Table. Data are presented as fold enrichment at the HO cut site (0.15 or 4.8 kb from the DSB) over that at the *PRE1* locus on chromosome V, then normalized to the corresponding input sample. The obtained fold enrichment values were normalized to the fold enrichment of the t_0_ sample. Standard mean error (SEM) was calculated on three independent experiments.

### Quantitative analysis of DSB end resection by real time PCR

Quantitative PCR (qPCR) analysis of DSB resection was performed accordingly to [52]. The oligonucleotides used are listed in S2 Table. The DNA was digested with the *Rsa*I restriction enzime (NEB) that cuts inside the amplicons at 0.15 kb and 4.8 kb from the DSB, but not in the *PRE1* control region on chromosome V. qPCR was performed on both digested and undigested templates using StoS Quantitative Master Mix 2X SYBR Green (Genespin) with the Biorad MyIQ2 PCR system. The ssDNA percentage over total DNA was calculated using the following formula: % ssDNA  =  *{100/[(1+2* ^Δ*Ct*^)/2]}/f, in which Δ*Ct* values are the difference in average cycles between digested template and undigested template of a given time point and *f* is the HO cut efficiency measured by Southern blot analysis.

### DSB end-tethering experiment

Cells of strains derivative from yJK40.6 background were grown in YEP+raf and blocked 3 hours in G2 with nocodazole. 160 µM CuSO_4_ was added one hour before inducing HO cut with galactose, accordingly to [58]. Samples taken at the indicated time were analysed with a fluorescence microscope. Cells with 2 LacI-GFP foci separated by more than 0.5 µm were considered defective in DSB end-tethering.

## Supporting Information

S1 Fig Deletion of *RAD9* rescues the lethality of the *sae2*Δ cells after a DSB through the helicase activity of Sgs1. (A) Meiotic tetrads from the indicated cross were dissected on YEPD plates that were incubated at 25°C, following by spores genotyping. (B) A plasmid vector expressing either the wild type or *sgs1*-K706A allele of *SGS1* gene was inserted by transformation into the YMV80 derivative *sae2*Δ *sgs1*Δ *rad9*Δ triple mutant. For each YMV80 derivative strain indicated in the Figure, the number of colonies grown after 3 days at 28°C in YEP+gal was normalized respect YEP+glu. Plotted values are the mean values ± SD from three independent experiments.
(TIF)

S2 Fig Deletion of *RAD9* rescues DSB repair defects of *sae2*Δ cells through a Rad51-independent pathway. (A) Exponentially growing cell cultures of the wild type YMV80 strain and the indicated derivatives were serially diluted (1∶10), and each dilution was spotted out into YEP+Raf or YEP+Raf+Gal plates. Plates were incubated 3 days at 28°C. (B) Exponentially growing YEP+raf cell cultures of the wild type YMV80 strain and the indicated derivatives were synchronized and kept blocked in G2/M phase with nocodazole treatment; galactose was added at time zero to induce HO-cut. Genomic DNA, extracted from samples taken at the indicated times, was analyzed for DSB formation and repair, as described in Fig. 2B.
(TIF)

S3 Fig Rad9 limits an Sgs1- and Exo1- dependent DSB resection. (A) Exponentially growing YEP+raf cell cultures of the wild type JKM139 strain and the indicated derivatives, carrying a unique HO cut site at *MAT* locus and expressing the HO nuclease under GAL1 promoter, were synchronized and kept in G2/M phases by nocodazole treatment. Galactose was added at time 0 to induce HO. Genomic DNA, extracted from samples taken at the indicated times, was analyzed for ssDNA formation, as described in Fig. 3B. (B) Densitometric analysis of the representative experiments shown in (A).
(TIF)

S4 Fig Analysis of DSB resection in *dot1*Δ derivative strains. (A) Exponentially growing YEP+raf cell cultures of the wild type JKM139 strain and the indicated derivatives, carrying a unique HO cut site at *MAT* locus and expressing the HO nuclease under GAL1 promoter, were synchronized and kept in G2/M phases by nocodazole treatment. Galactose was added at time 0 to induce HO. Genomic DNA, extracted from samples taken at the indicated times, was analyzed for ssDNA formation, as described in Fig. 3B. Wild type and *sae2*Δ blots are the same used in Fig. 3B. (B) Densitometric analysis of the representative experiments shown in (A). (C) Plot showing the ratio of resected DNA among HO cut DNA at each time points by qPCR analysis, measured at 0.15 kb as described in Fig. 3D.
(TIF)

S5 Fig Analysis of Rad9 binding near a DSB. Cells of the wild type JKM139 strain and the indicated derivatives, expressing a Rad9-3HA fusion protein, were grown in YEP+raf and synchronized in G2/M phases by nocodazole treatment. Galactose was added at time 0 to induce HO. Relative fold enrichment of Rad9-3HA at 0.1 kb from the HO cleavage site was evaluated after ChIP with anti-HA antibodies and qPCR analysis. Plotted values are the mean values ± SEM from three independent experiments.
(TIF)

S6 Fig Analysis of the expression levels and phosphorylation of various Rad9 protein variants. (A) Cells of the wild type YMV80 strain and the indicated derivatives were grown in YEP+raf. Galactose was added at time 0 to induce HO. Cells have been taken at the indicated times and protein extracts were done. Rad9 and Rad53 were detected by western blotting. (B) Cells of the wild type YMV80 strain and the *rad9*-ΔBRCT-FKBP derivative were grown in YEP+raf. Cell cultures were split in two and one half was treated with AP20187 for 1 hr, before adding galactose to induce HO. Cells have been taken at the indicated times and protein extracts were done. Rad9 and Rad53 were detected by western blotting.
(TIF)

S7 Fig Deletion of *KU70* does not rescue viability of YMV80 derivative *sae2*Δ cells, following a DSB. Viability of the wild type YMV80 strain and the indicated derivatives, plated on YEP+gal. For each strain, the number of colonies grown after 3 days at 28°C in YEP+gal was normalized respect YEP+glu. Plotted values are the mean values ± SD from three independent experiments.
(TIF)

S1 Table List of yeast strains described in this work.
(DOCX)

S2 Table List of the oligonucleotides used for ChIP and DSB resection analyses.
(DOCX)
