# Supplementary figures and images for: Functional Interplay between the 53BP1-Ortholog Rad9 and the Mre11 Complex Regulates Resection, End-Tethering and Repair of a Double-Strand Break
Source: PLoS Genet. 2015 Jan 8;11(1):e1004928. doi: 10.1371/journal.pgen.1004928 (PMC4287487; doi:10.1371/journal.pgen.1004928)

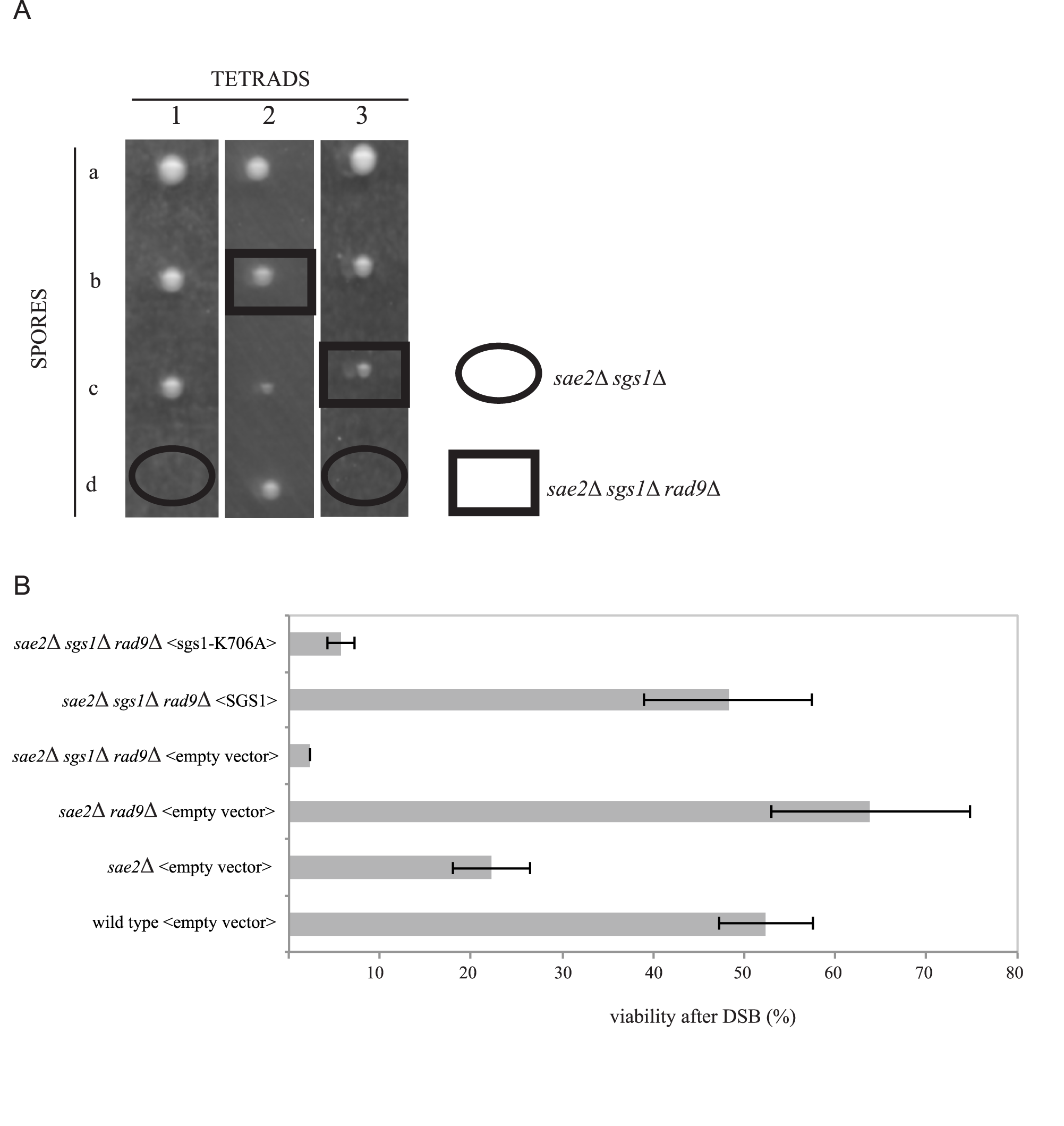

Supplement: S1 Fig — Deletion of RAD9 rescues the lethality of the sae2Δ cells after a DSB through the helicase activity of Sgs1. (A) Meiotic tetrads from the indicated cross were dissected on YEPD plates that were incubated at 25°C, following by spores genotyping. (B) A plasmid vector expressing either the wild type or sgs1-K706A allele of SGS1 gene was inserted by transformation into the YMV80 derivative sae2Δ sgs1Δ rad9Δ triple mutant. For each YMV80 derivative strain indicated in the Figure, the number of colonies grown after 3 days at 28°C in YEP+gal was normalized respect YEP+glu. Plotted values are the mean values ± SD from three independent experiments. (TIF) [file pgen.1004928.s001.tif]

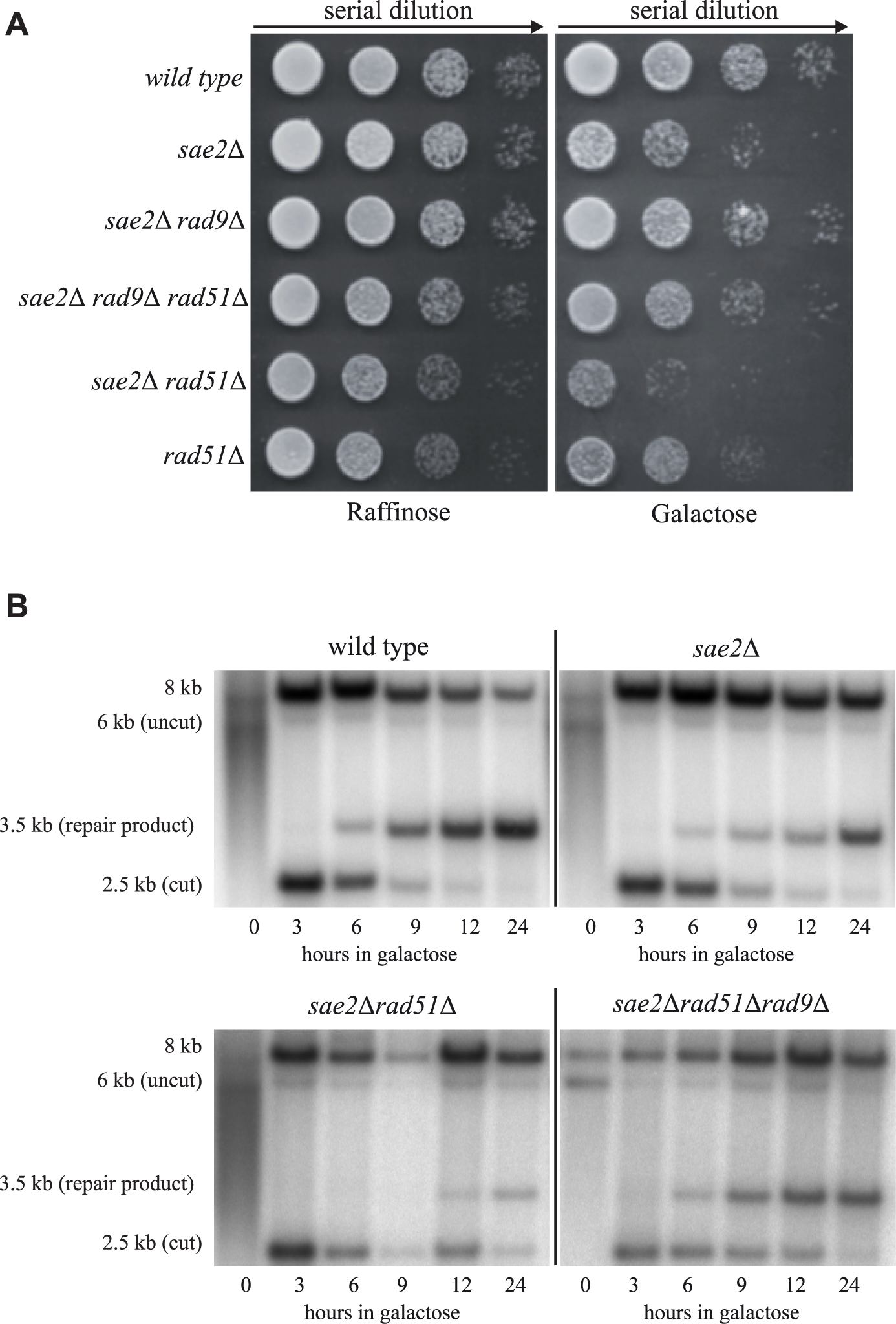

Supplement: S2 Fig — Deletion of RAD9 rescues DSB repair defects of sae2Δ cells through a Rad51-independent pathway. (A) Exponentially growing cell cultures of the wild type YMV80 strain and the indicated derivatives were serially diluted (1∶10), and each dilution was spotted out into YEP+Raf or YEP+Raf+Gal plates. Plates were incubated 3 days at 28°C. (B) Exponentially growing YEP+raf cell cultures of the wild type YMV80 strain and the indicated derivatives were synchronized and kept blocked in G2/M phase with nocodazole treatment; galactose was added at time zero to induce HO-cut. Genomic DNA, extracted from samples taken at the indicated times, was analyzed for DSB formation and repair, as described in Fig. 2B. (TIF) [file pgen.1004928.s002.tif]

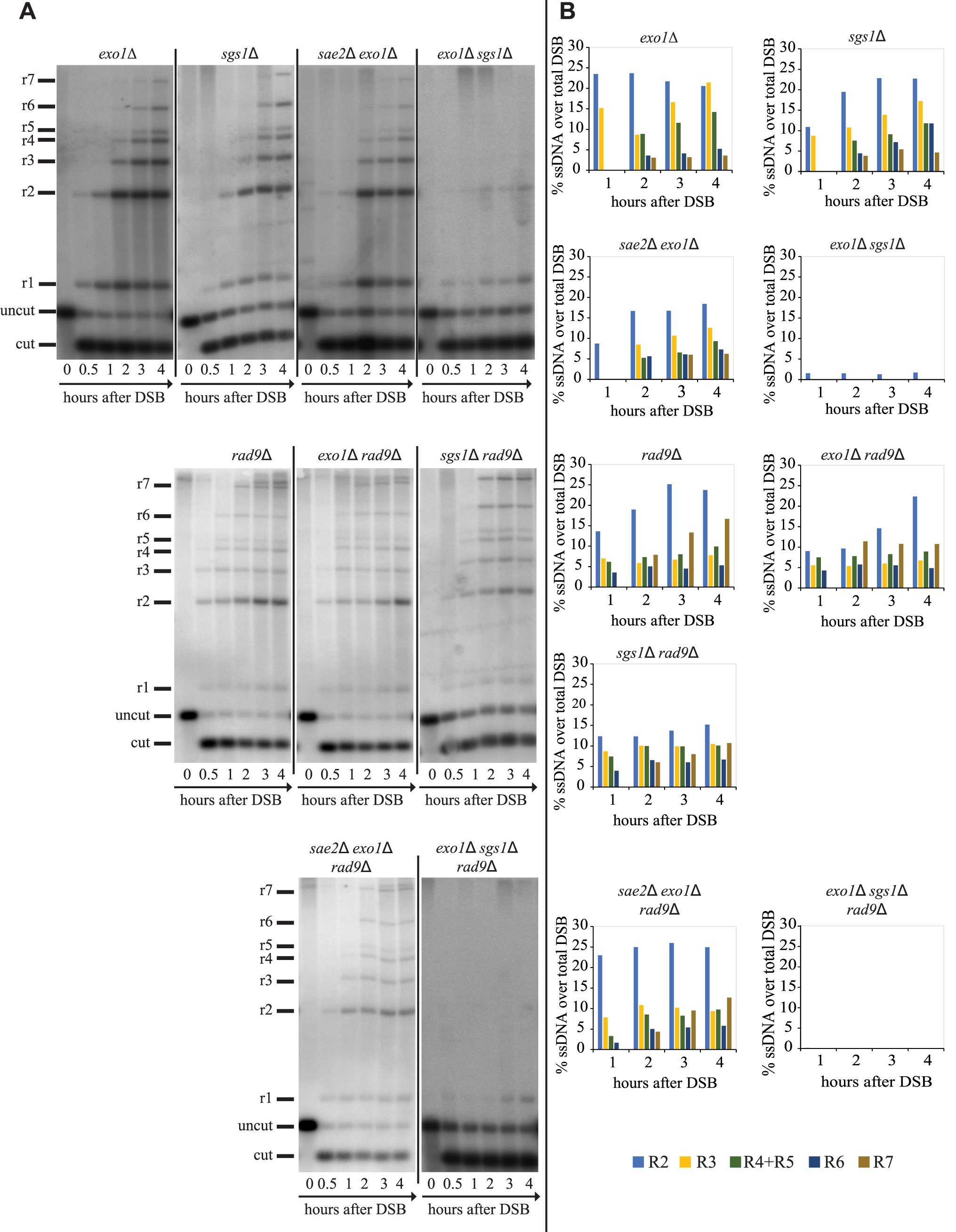

Supplement: S3 Fig — Rad9 limits an Sgs1- and Exo1- dependent DSB resection. (A) Exponentially growing YEP+raf cell cultures of the wild type JKM139 strain and the indicated derivatives, carrying a unique HO cut site at MAT locus and expressing the HO nuclease under GAL1 promoter, were synchronized and kept in G2/M phases by nocodazole treatment. Galactose was added at time 0 to induce HO. Genomic DNA, extracted from samples taken at the indicated times, was analyzed for ssDNA formation, as described in Fig. 3B. (B) Densitometric analysis of the representative experiments shown in (A). (TIF) [file pgen.1004928.s003.tif]

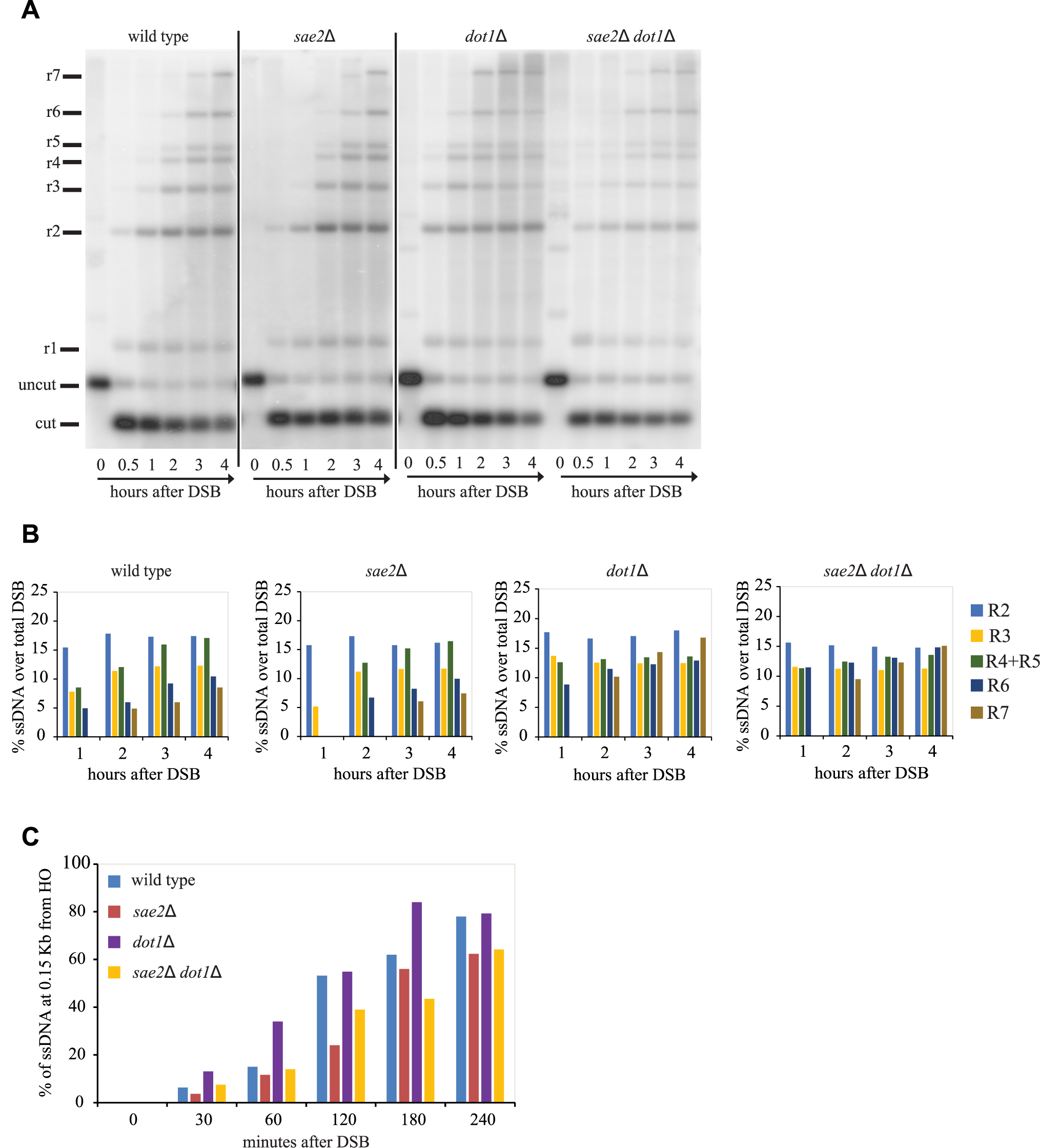

Supplement: S4 Fig — Analysis of DSB resection in dot1Δ derivative strains. (A) Exponentially growing YEP+raf cell cultures of the wild type JKM139 strain and the indicated derivatives, carrying a unique HO cut site at MAT locus and expressing the HO nuclease under GAL1 promoter, were synchronized and kept in G2/M phases by nocodazole treatment. Galactose was added at time 0 to induce HO. Genomic DNA, extracted from samples taken at the indicated times, was analyzed for ssDNA formation, as described in Fig. 3B. Wild type and sae2Δ blots are the same used in Fig. 3B. (B) Densitometric analysis of the representative experiments shown in (A). (C) Plot showing the ratio of resected DNA among HO cut DNA at each time points by qPCR analysis, measured at 0.15 kb as described in Fig. 3D. (TIF) [file pgen.1004928.s004.tif]

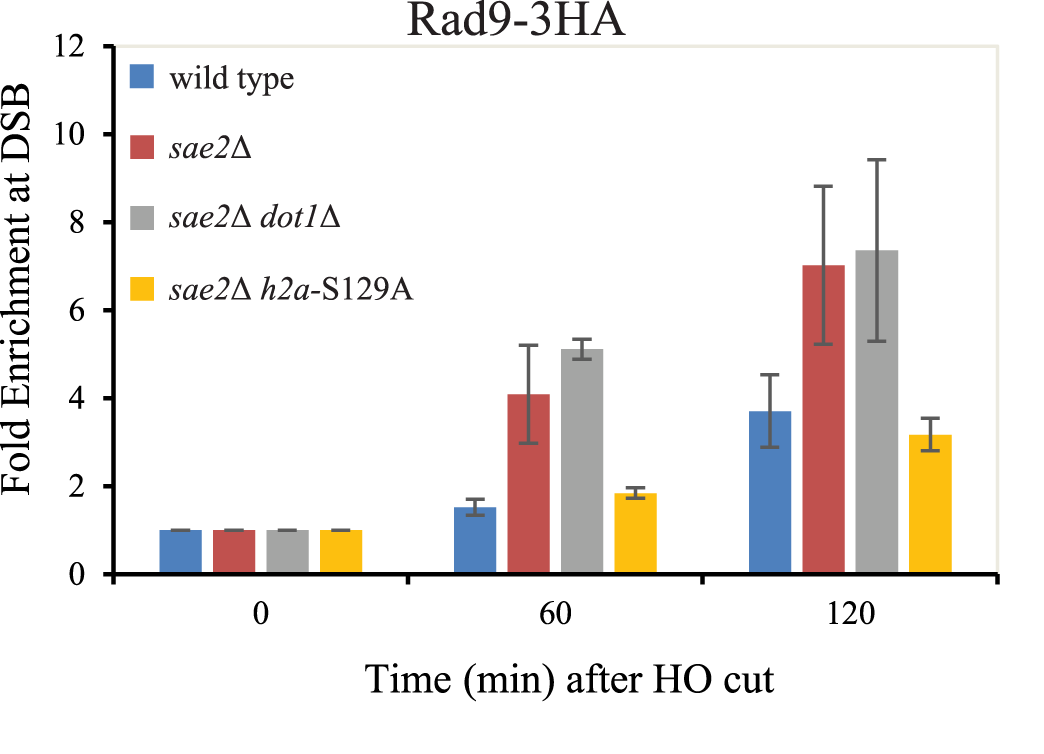

Supplement: S5 Fig — Analysis of Rad9 binding near a DSB. Cells of the wild type JKM139 strain and the indicated derivatives, expressing a Rad9-3HA fusion protein, were grown in YEP+raf and synchronized in G2/M phases by nocodazole treatment. Galactose was added at time 0 to induce HO. Relative fold enrichment of Rad9-3HA at 0.1 kb from the HO cleavage site was evaluated after ChIP with anti-HA antibodies and qPCR analysis. Plotted values are the mean values ± SEM from three independent experiments. (TIF) [file pgen.1004928.s005.tif]

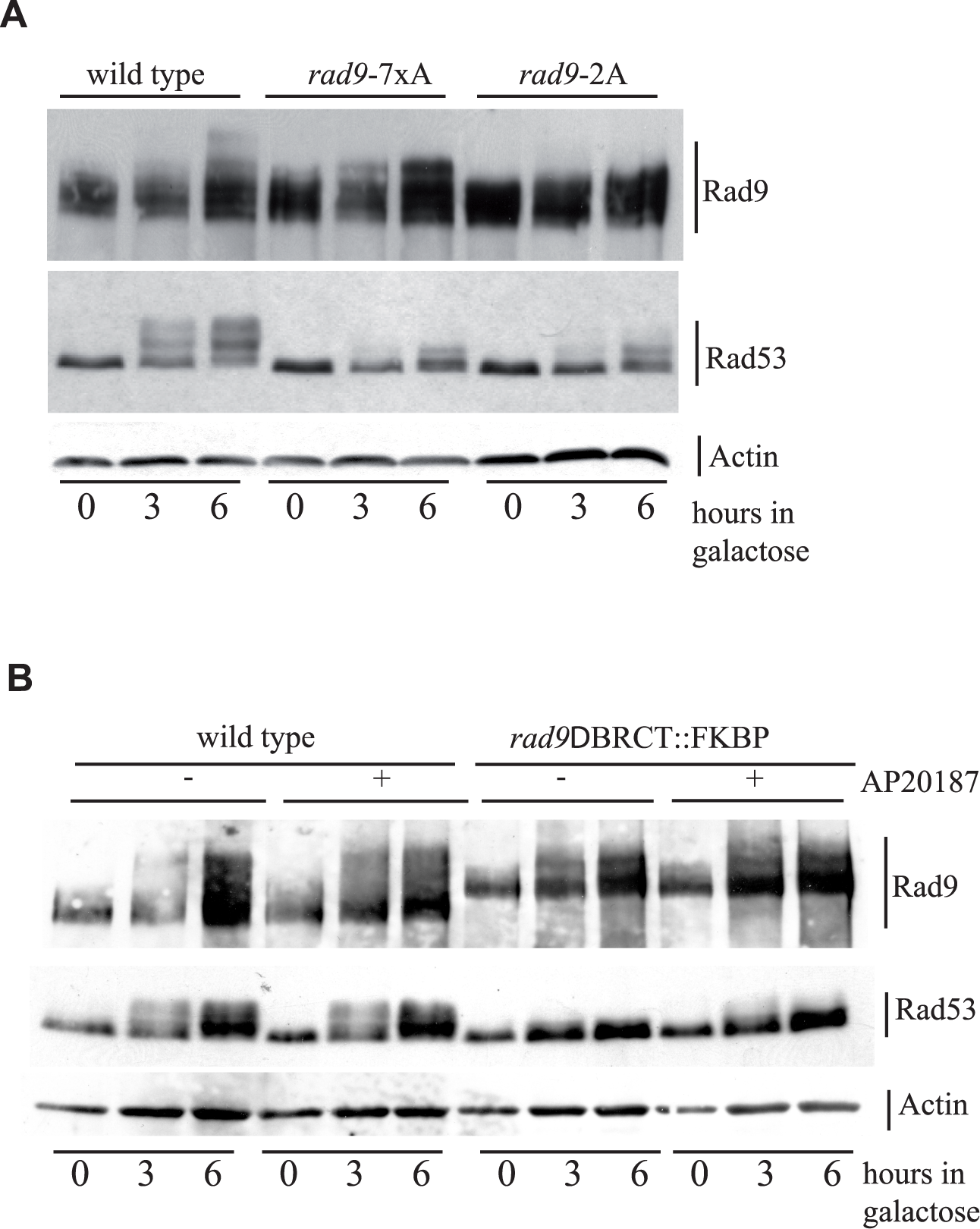

Supplement: S6 Fig — Analysis of the expression levels and phosphorylation of various Rad9 protein variants. (A) Cells of the wild type YMV80 strain and the indicated derivatives were grown in YEP+raf. Galactose was added at time 0 to induce HO. Cells have been taken at the indicated times and protein extracts were done. Rad9 and Rad53 were detected by western blotting. (B) Cells of the wild type YMV80 strain and the rad9-ΔBRCT-FKBP derivative were grown in YEP+raf. Cell cultures were split in two and one half was treated with AP20187 for 1 hr, before adding galactose to induce HO. Cells have been taken at the indicated times and protein extracts were done. Rad9 and Rad53 were detected by western blotting. (TIF) [file pgen.1004928.s006.tif]

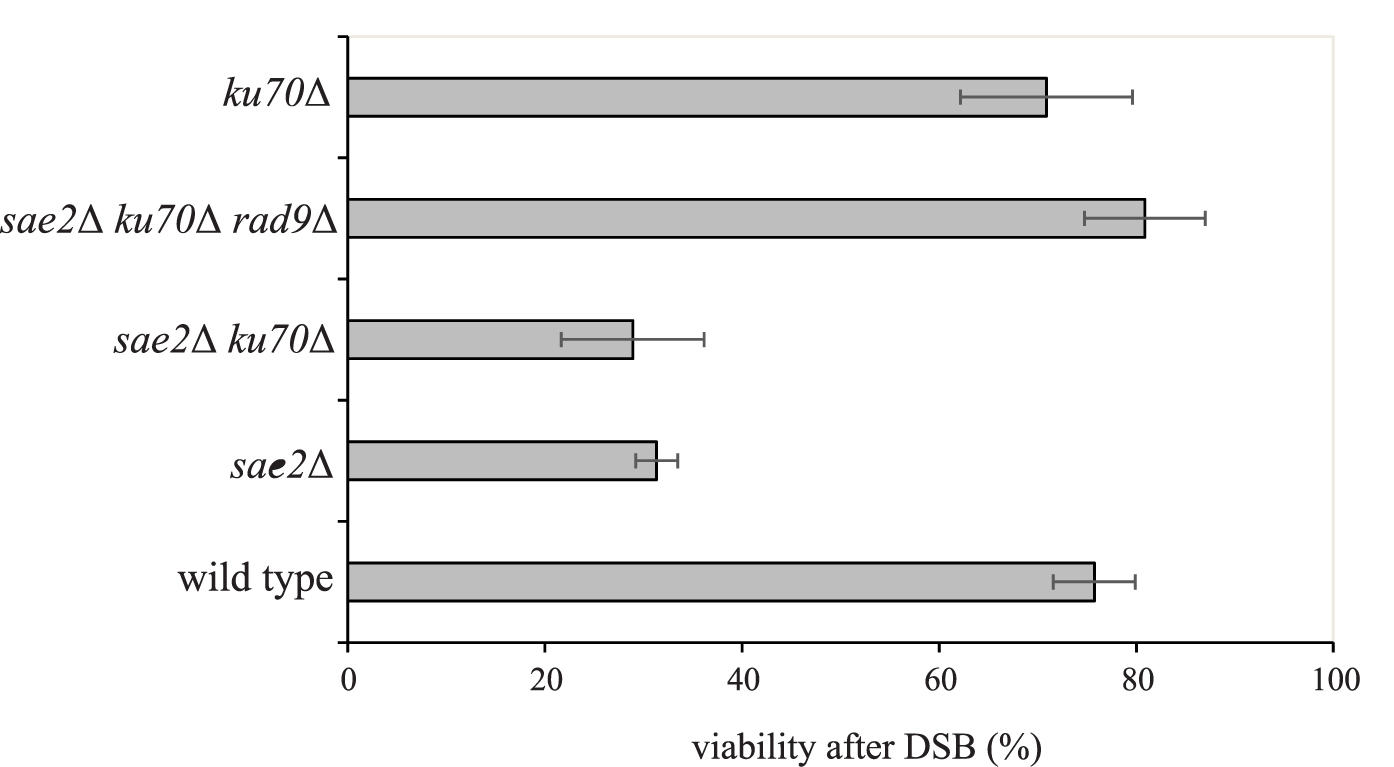

Supplement: S7 Fig — Deletion of KU70 does not rescue viability of YMV80 derivative sae2Δ cells, following a DSB. Viability of the wild type YMV80 strain and the indicated derivatives, plated on YEP+gal. For each strain, the number of colonies grown after 3 days at 28°C in YEP+gal was normalized respect YEP+glu. Plotted values are the mean values ± SD from three independent experiments. (TIF) [file pgen.1004928.s007.tif]
