# Supplementary material for: Functional Interplay between the 53BP1-Ortholog Rad9 and the Mre11 Complex Regulates Resection, End-Tethering and Repair of a Double-Strand Break
Source: PLoS Genet. 2015 Jan 8;11(1):e1004928. doi: 10.1371/journal.pgen.1004928 (PMC4287487; doi:10.1371/journal.pgen.1004928)
Supplement: S1 Table — List of yeast strains described in this work. (DOCX) [file pgen.1004928.s008.docx]

**Table S1. List of yeast strains described in this work.**

| Strain  name | Parental strain | Genotype | Source |
| --- | --- | --- | --- |
| Y1600 | JKM139 | *MATa ho hml::ADE1 hmr::ADE1 ade1-100 leu2-3, 112 lys5, trp1::hisG ura3-52 lys5::ade3::GAL10::HO* | Lee et al., 1998 |
| Y1126 | JKM179 | *MATalpha ho hml::ADE1 hmr::ADE1 ade1-100 leu2-3, 112 lys5, trp1::hisG ura3-52 lys5::ade3::GAL10::HO* | Lee et al., 1998 |
| Y92 | Y1126 | *MATalpha sae2::KANMX6 ku70::URA3 exo1::TRP1* | This Study |
| Y95 | Y1126 | *MATalpha sae2::KANMX6* | This study |
| Y500 | JKM139 | *MRE11-18MYC::TRP1* | Clerici et al., 2006 |
| Y602 | Y1600 | *MATa rad9::KANMX6* | This study |
| Y642 | Y1600 | *MATa sae2::KANMX6* | This study |
| YFL489 | Y1600 | *MATa dot1::KANMX6* | Lazzaro et al., 2008 |
| Y1813 | Y92 X YFL489 | *MATa sae2::KANMX6 exo1::TRP1* | This study |
| Y1853 | Y92 X Y602 | *MATa exo1::TRP1* | This study |
| Y1856 | Y92 X Y602 | *MATalpha sae2::KANMX6 rad9::KANMX6* | This study |
| Y1858 | Y92 X Y602 | *MATa sae2::KANMX6 rad9::KANMX6* | This study |
| Y1860 | Y92 x Y602 | *MATa rad9::KANMX6 exo1::TRP1* | This study |
| Y1861 | Y92 X Y602 | *MATa sae2::KANMX6 rad9::KANMX6 exo1::TRP1* | This study |
| Y1863 | Y92 X Y602 | *MATa sae2::KANMX6 rad9::KANMX6 exo1::TRP1 ku70::URA3* | This study |
| Y1866 | Y1126 | *MATalpha sgs1::HPH* | This study |
| Y1879 | Y602 X Y1866 | *MATa rad9::KANMX6 sgs1::HPH* | This study |
| Y1883 | Y1863 X Y1866 | *MATa sgs1::HPH* | This study |
| Y1910 | Y1858 X Y1866 | *MATa sae2::KANMX6 rad9::KANMX6 sgs1::HPH* | This study |
| Y2137 | Y500 X Y1856 | *MATa sae2::KANMX6 rad9::KANMX6 MRE11-18MYC::TRP1* | This study |
| Y2140 | Y1600 | *MATa sae2::KANMX6 MRE11-18Myc::TRP1* | This study |
| Y2141 | Y1600 | *MATa rad9::KANMX6 MRE11-18MYC::TRP1* | This study |
| Y2142 | Y500 X Y1856 | *MATalpha rad9::KANMX6 MRE11-18MYC::TRP1* | This study |
| Y2197 | Y1860 X Y1883 | *MATa exo1::TRP1 rad9::KANMX6 sgs1::HPH* | This study |
| Y2200 | Y1860 X Y1883 | *MATa exo1::TRP1 sgs1::HPH* | This study |
| Y2213 | Y2044 | *MATa rad9::HPH RAD9::3XHA::URA3::ura3-52* | This study |
| Y2241 | Y95 X Y2213 | *MATa sae2::KANMX6 rad9::HPH RAD9::3XHA::URA3::ura3-52* | This study |
| Y2254 | Y1883 | *MATa sgs1::HPH MRE11-18MYC::TRP1* | This study |
| Y2266 | Y95 X YFL489 | *MATa sae2::KANMX6 dot1::KANMX6* | This study |
| Y2267 | Y95 X YFL489 | *MATalpha sae2::KANMX6 dot1::KANMX6* | This study |
| Y2312 | Y1910 X Y2142 | *MATa sae2::KANMX6 rad9::KANMX6 sgs1::HPH MRE11-18MYC::TRP1* | This study |
| Y2537 | Y602 | *MATa rad9::KANMX6 rad9::2A::URA3* | This study |
| Y2634 | Y2537 | *MATa sae2::HPH rad9::KANMX6 rad9::2A::URA3* | This study |
| Y2664 | Y1600 | *MATa RAD9::3XHA::KANMX6* | This study |
| Y2665 | Y2267 X Y2664 | *MATa sae2::KANMX6 RAD9::3XHA::KANMX6* | This study |
| Y2671 | Y2264 X Y2267 | *MATa sae2::KANMX6 dot1::KANMX6 RAD9::3XHA::KANMX6* | This study |
| R726 | Y1600 | *MATa hta1-S129A hta2-S129A* | Haber’s stock |
| Y2707 | R726 | *MATa hta1-S129A hta2-S129A RAD9::3XHA::KANMX6* | This study |
| Y2723 | Y2707 | *MATa hta1-S129A hta2-S129A sae2::KANMX6 RAD9::3XHA::KANMX6* | This study |
| Y1601 | YMV80 | *ho hml ::ADE1 mata ::hisG hmr ::ADE1 his4::NatMXleu2-(XhoI- to Asp718) leu2::MATa ade3::GAL::HO ade1 lys5 ura3-52 trp1* | Vaze et al., 2002 |
| Y28 | Y1601 | *exo1::KANMX6* | This study |
| Y78 | Y1601 | *sae2::KANMX6* | This study |
| Y1270 | Y1601 | *ADH::TIR1::URA3* | This study |
| Y1526 | Y1601 | *rad9::HPH* | This study |
| Y1570 | Y1601 | *sae2::KANMX6 rad9::HPH* | This study |
| Y1646 | Y1601 | *sae2::KANMX6 dot1::TRP1* | This study |
| Y1671 | Y1601 | *mre11-D56N* | This study |
| Y1744 | Y1601 | *mre11-D56N rad9::HPH* | This study |
| Y1799 | Y1601 | *sae2::HPH rad9::TRP1 exo1::KANMX6* | This study |
| Y1953 | Y1601 | *sgs1::HPH* | This study |
| Y1961 | Y1601 | *sgs1::HPH rad9::TRP1* | This study |
| Y1967 | Y1601 | *sgs1::HPH rad9::TRP1 sae2::KANMX6* | This study |
| Y2016 | Y1601 | *exo1::KANMX6 sgs1::HPH rad9::TRP1* | This study |
| Y2044 | Y1600 | *rad9::HPH* | This study |
| Y2101 | Y1270 | *dna2::DEG::KANMX6 ADH::TIR1::URA3* | This study |
| Y2127 | Y1601 | *sae2::KANMX6 rad9::HPH RAD9-7XA::URA3* | This study |
| Y2131 | Y1601 | *rad9::HPH RAD9-7XA::URA3* | This study |
| Y2136 | Y1601 | *hta1-S129A* | J Haber’s lab |
| Y2172 | Y1601 | *sae2::KANMX6 hta1-S129A* | This study |
|  |  |  |  |
| Y2214 | Y1601 | *mre11-D56N rad9::HPH sae2::KANMX6* | This study |
| Y2236 | Y2101 | *sae2::HPH rad9::TRP1 dna2::DEG::KANMX6 ADH::TIR1::URA3* | This study |
| Y2308 | Y1601 | *rad9::HPH RAD9-S462A,T474A::URA3* | This study |
| Y2310 | Y1601 | *sae2::KANMX6 rad9::HPH RAD9-S462A,T474A::URA3* | This study |
| Y2365 | Y1601 | *mre11-D56N rad9::HPH sgs1::KANMX6* | This study |
| Y2384 | Y1601 | *rad9ΔBRCT-FKBP2x-13MYC::KANMX6* | This study |
| Y2385 | Y1601 | *sae2::HPH rad9ΔBRCT-FKBP2x-13MYC::KANMX6* | This study |
| Y2387 | Y1601 | *chk1::TRP1 rad53-K227A::KANMX6* | This study |
| Y2388 | Y1601 | *sae2::HPH chk1::TRP1 rad53-K227A::KANMX6* | This study |
| Y2390 | Y1601 | *exo1::KANMX6 sgs1::HPH* | This study |
| Y2550 | Y1601 | *sgs1::HPH rad9::TRP1 sae2::KANMX6 + [SGS1]* | This study |
| Y2551 | Y1601 | *sgs1::HPH rad9::TRP1 sae2::KANMX6 + [sgs1-hd]* | This study |
| Y2731 | Y1601 | *rad50::HPH* | Lab’s stock |
| Y2732 | Y2731 | *rad50::HPH sae2::KANMX6* | This study |
| YFL804 | Y1601 | *rad50::HPH rad9::KANMX6* | Lazzaro et al., 2008 |
| YFL736 | Y1601 | *dot1::KANMX6* | Lazzaro et al., 2008 |
| YFL802 | Y1601 | *exo1::KANMX6 rad9::TRP1* | Lazzaro et al., 2008 |
| YFL809 | Y1601 | *rad50::KANMX6* | Lazzaro et al., 2008 |
| YFL827 | Y1601 | *rad50::KANMX6 rad9::HPH* | Lazzaro et al., 2008 |
| Y502 | YJK40.6 | *MAT∆ hml∆ hmr∆ can1 lys5 ade2 leu2 trp1 ura3 his3 ade3::GAL-HO VII::TRP1-HO LacI-GFP::URA3 LacO::LYS5 LacO::KanR* | Kaye et al., 2004 |
| Y1244 | YJK40.6 | *sae2::HPH* | This study |
| Y2356 | YJK40.6 | *sae2::HPH rad9::NAT* | This study |
| Y2539 | YJK40.6 | *sae2::HIS sgs1::HPH rad9::NAT* | This study |
