# Supplementary material for: Functional Interplay between the 53BP1-Ortholog Rad9 and the Mre11 Complex Regulates Resection, End-Tethering and Repair of a Double-Strand Break
Source: PLoS Genet. 2015 Jan 8;11(1):e1004928. doi: 10.1371/journal.pgen.1004928 (PMC4287487; doi:10.1371/journal.pgen.1004928)
Supplement: S2 Table — List of the oligonucleotides used for ChIP and DSB resection analyses. (DOCX) [file pgen.1004928.s009.docx]

**Table S2. List of the oligonucleotides used for ChIP and DSB resection analyses.**

| Name | Sequence (5’-3’) | Distance from DSB | Source |
| --- | --- | --- | --- |
| QMAT1F | CCTGGTTTTGGTTTTGTAGAGTGG | 0,15 Kb (Chr III) | Kim et al. 2007 |
| QMAT1R | GAGCAAGACGATGGGGAGTTTC | 0,15 Kb (ChrIII) | Kim et al. 2007 |
| QMAT2F | ATTGCGACAAGGCTTCACCC | 4,8 kb (ChrIII) | Kim et al. 2007 |
| QMAT2R | CCACATCACAGGTTTATTGGTTCC | 4,8 kb (ChrIII) | Kim et al. 2007 |
| QPRE1F | CCCACAAGTCCTCTGATTTACATTCG | Chr V | Shim et al. 2005 |
| QPRE1R | ATTCGATTGACAGGTGCTCCCTTTTC | Chr V | Shim et al. 2005 |
